# SLPI facilitates cell migration by regulating lamellipodia/ruffles and desmosomes, in which Galectin4 plays an important role

**DOI:** 10.1080/19336918.2020.1829264

**Published:** 2020-10-04

**Authors:** Yusuke Mizutani, Daisuke Omagari, Manabu Hayatsu, Masaaki Nameta, Kazuo Komiyama, Yoshikazu Mikami, Tatsuo Ushiki

**Affiliations:** a Division of Microscopic Anatomy, Niigata University Graduate School of Medical and Dental Sciences, Niigata-shi, Japan; b Office of Institutional Research, Hokkaido University, Kita-ku, Japan; c Department of Pathology, Nihon University School of Dentistry, Tokyo, Japan; d Electron Microscope Core Facility, Niigata University, Niigata-shi, Japan

**Keywords:** Carcinoma, SLPI, migration, microscopic analysis, Galectin4

## Abstract

To elucidate the underlying mechanism of secretory leukocyte protease inhibitor (SLPI)-induced cell migration, we compared SLPI-deleted human gingival carcinoma Ca9-22 (ΔSLPI) cells and original (wild-type: wt) Ca9-22 cells using several microscopic imaging methods and gene expression analysis. Our results indicated reduced migration of ΔSLPI cells compared to wtCa9-22 cells. The lamellipodia/dorsal ruffles were smaller and moved slower in ΔSLPI cells compared to wtCa9-22 cells. Furthermore, well-developed intermediate filament bundles were observed at the desmosome junction of ΔSLPI cells. In addition, *Galectin4* was strongly expressed in ΔSLPI cells, and its forced expression suppressed migration of wtCa9-22 cells. Taken together, SLPI facilitates cell migration by regulating lamellipodia/ruffles and desmosomes, in which Galectin4 plays an important role.

## Introduction

Secretory leukocyte protease inhibitor (SLPI) is an important regulator of innate and adaptive immunity [1,2]. Alternatively, SLPI plays a role in cancer cell malignancy and is upregulated in various types of cancer cells [3–6]. SLPI is upregulated in highly malignant Lewis lung carcinoma (LLC) cells compared to that in less malignant LLC cells [7]. Furthermore, forced expression of SLPI in human breast cancer cell lines programs cells for vascular mimicry, thereby contributing to distant metastases [8]. We previously reported similar results in a gingival carcinoma Ca9-22 cell line expressing high levels of SLPI [9]. Comparative analysis of the invasiveness using a myoma tissue culture model mimicking the native human tumor microenvironment indicated the formation of a thick cellular layer over the myoma tissue by SLPI-deleted (ΔSLPI) Ca9-22 cells, while Ca9-22 cells invaded the myoma tissue. However, the mechanism underlying SLPI-induced enhancement of the malignant phenotype remains unclear.

Malignancy of cancer cells depends on the potential for cell migration [10]. Specific protrusive and contractile actin filaments, called lamellipodia and filopodia, drive cell migration [11]. Furthermore, cell migration is partly regulated through cell adhesion [12]. Actin cytoskeleton or intermediate filaments stabilize cell-cell and cell-extracellular matrix (ECM) adhesion complexes. However, these are dynamically rearranged under certain circumstances including cell migration and cancer metastasis [13,14].

This study aimed to elucidate the mechanism underlying SLPI-induced enhancement of the malignant phenotype by characterizing the morphology of both the actin filament and cell adhesion structures in SLPI-expressing cancer cells and to identify the molecules associated with the process.

## Results

**Figure 1.**
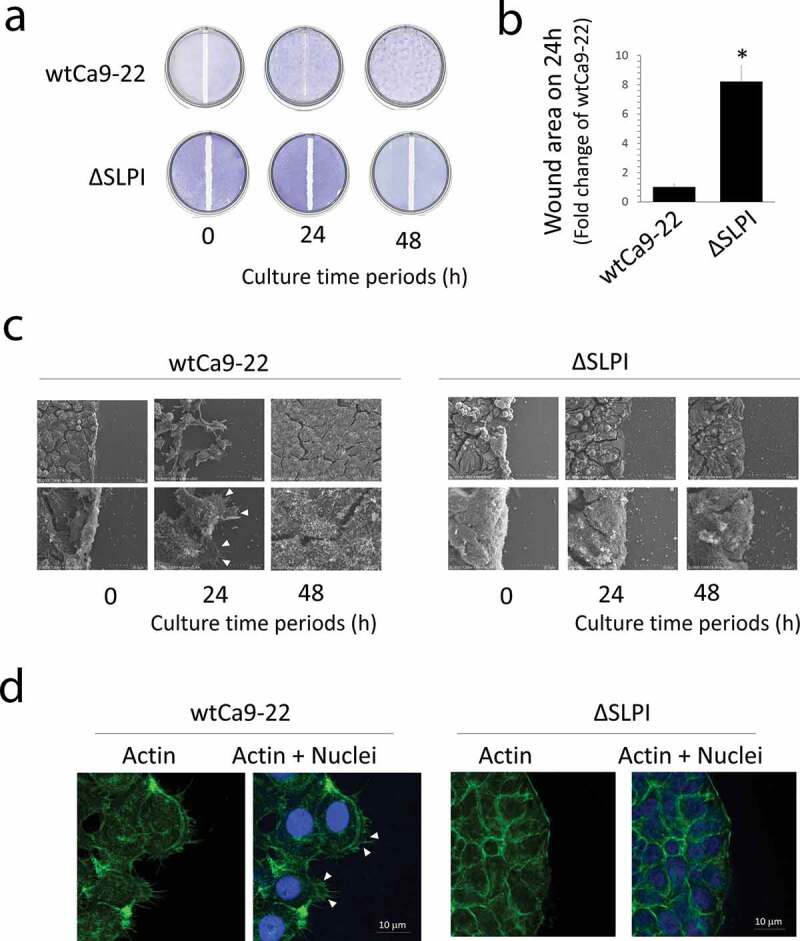
(a) A scratch ‘wound’ was made using a silicone tip at the center of a confluent monolayer culture and the cells were further cultured for the indicated duration. The cell layers were fixed and stained with toluidine blue. (b) Cell migration was quantified in the wound areas 24 h after injury. Quantitative data were presented as the mean ± standard deviation (n = 3, *P < 0.05 vs. wtCa9-22). (c) Cells were cultured under the same conditions as in (a) and were observed using SEM. The arrow heads indicate the lamellipodia. (c) Histochemical staining of wtCa9-22 and ΔSLPI cells highlighting the original scratch zone. Cells were cultured under the same conditions as in (A). (d) Histochemical staining was performed using Alexa Fluor^TM^ 488 phalloidin (green). Nuclei were stained with DAPI (blue). Arrow heads indicate the lamellipodia.

The *in vitro* wound healing assay indicated lower migration of ΔSLPI cells from the border of the original scratch zone when compared with wtCa9-22 cells, confirming the previously described results [9] (Figure 1(a)). These observations were supported by quantitative data determined from around wound areas (Figure 1(b)). Further, we observed cell migration under standard culture conditions (un-confluent), exhibiting similar results to that of the *in vitro* wound healing assay. WtCa9-22 cells highly migrated, and in contrast, ΔSLPI cells scarcely migrated (Suppl. Video S1 online). Cellular microstructures in the scratch zone border (shown in A) were analyzed thereafter. SEM and histochemical assessment of cells stained with Alexa FluorTM 488 phalloidin, which binds to actin filaments, revealed the presence of elongated lamellipodia with actin filaments in wtCa9-22 cells. In contrast, lamellipodia were scarcely detected in ΔSLPI cells (Figure 1(c,d)).

**Figure 2.**
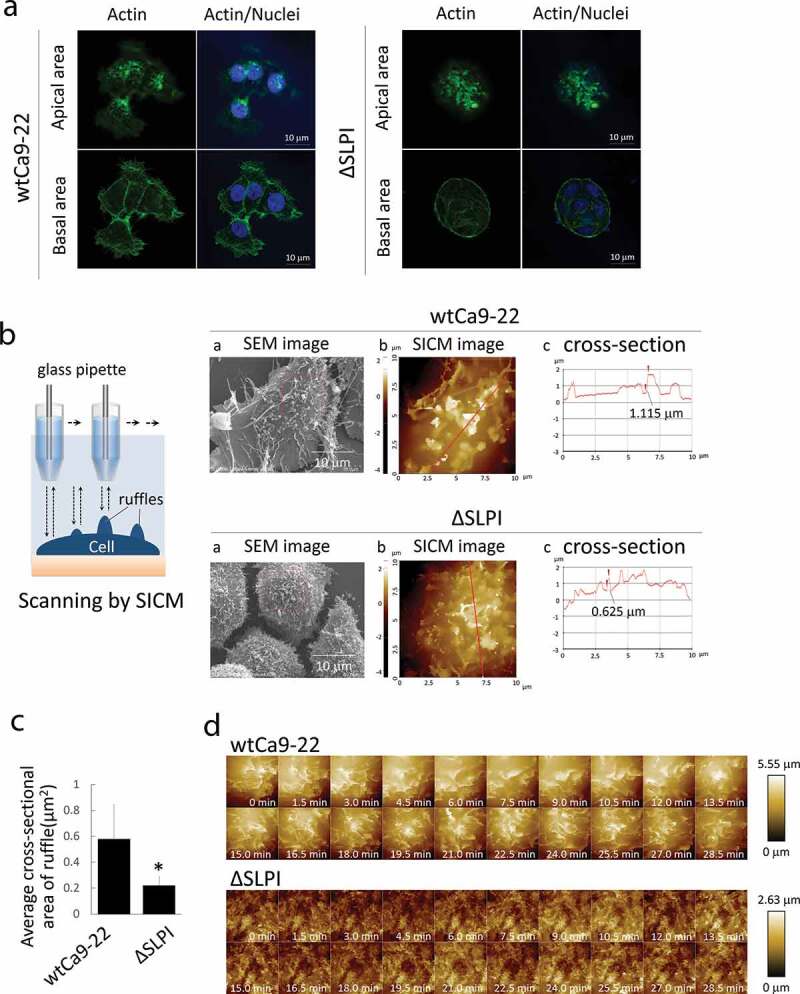
(a) Cells in the growth phase were stained with Alexa Fluor^TM^ 488 phalloidin (green). Nuclei were stained with DAPI (blue). (b) A schematic representation of SICM scanning. (a) SEM images of wtCa9-22 cells. The red square indicates the image area in (b). (b) SICM topographic images obtained at 10 × 10 μm^2^ with 128 × 128 pixels. (c) Cross-sectional graphs of these cells are included with a red line on the maximum diameter in each cell in (b). Numerals in the graph indicate the size of height of the dorsal ruffle indicated by arrowheads. (c) Quantification of the dorsal ruffle area was performed. Five cells were randomly selected from each cell type, and the average cross-sectional ruffle area was calculated. (d) Time-lapse imaging of wtCa9-22 (upper) and ΔSLPI cell (lower) surface. To assess ruffle movement, time-lapse morphological images of the ruffles on the surface of the living cells were obtained through SICM. All images were obtained at 10 × 10 μm^2^ with 64 × 64 pixels.

Phalloidin staining was performed to assess the ruffle structures with actin filaments in both apical and basal areas in each cell type cultured under normal conditions. Similar to Figure 1(d), elongated lamellipodia with actin filaments were observed in wtCa9-22 cells but not in ΔSLPI cells in the basal area. Conversely, ruffle structures with actin filaments were observed in both cell types in the apical area, although detailed structures were not revealed (Figure 2(a)). Thus, for detailed investigation, SEM images and SICM analysis were performed, and cross-sectional images of wtCa9-22 and ΔSLPI cells are presented in Figure 2(b). Comparative morphological analysis revealed larger dorsal ruffles ranging 100 nm to 1 μm on the surface of wtCa9-22 cells compared to that of ΔSLPI cells, exhibiting smaller dorsal ruffles, indicating that the network of actin filaments were associated with ruffle movement. These observations were supported by quantification of cross-sectional area of dorsal ruffles (Figure 2(c)). Furthermore, to assess the movement of the dorsal ruffles, time-lapse morphological images of the ruffles on the surface of living cells were obtained through SICM (Figure 2(d), Suppl. Video S2 online). Time-lapse imaging clearly indicated constant movement of the ruffles. The dorsal ruffles were large and rapidly moving in wtCa9-22 cells compared to that observed in ΔSLPI cells, wherein the dorsal ruffles were small and moved gradually in different directions.

**Figure 3.**
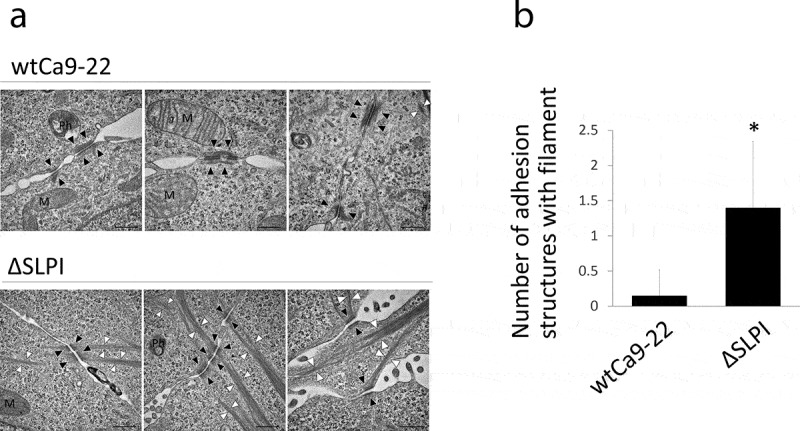
(a) Typical TEM images of wtCa9-22 and ΔSLPI cells in a cluster. Well-developed filaments elongating from the desmosome junction are observed in the cytoplasm of ΔSLPI cells in the cluster. N: nucleus; M: mitochondria; Ph: phagosome; arrowhead (white): cytoplasmic filament bundle; arrowhead (black): desmosome junction. Scale bar: 0.5 µm. (b) Quantification of the number of desmosome junctions with filament bundles. The number of desmosome junctions with filament bundles was determined in 20 sets of cell-cell adhesion structures in wtCa9-22 and ΔSLPI cell clusters, respectively, and the average mean ± standard deviation was determined (n = 20, *P < 0.05 vs. wtCa9-22).

TEM analysis was performed to examine the intracellular and cell-cell adhesion structures present in wtCa9-22 and ΔSLPI cells. Representative electron micrographs of wtCa9-22 and ΔSLPI cells are displayed in Figure 3(a). In wtCa9-22 cells, most cytoplasmic filaments were detected as network-shaped structures near desmosome junctions. In contrast, well-developed cytoplasmic filament bundles elongating from the desmosome junction into the cytoplasm were detected in ΔSLPI cells. These observations were further supported by TEM quantification data (Figure 3(b)).

**Figure 4.**
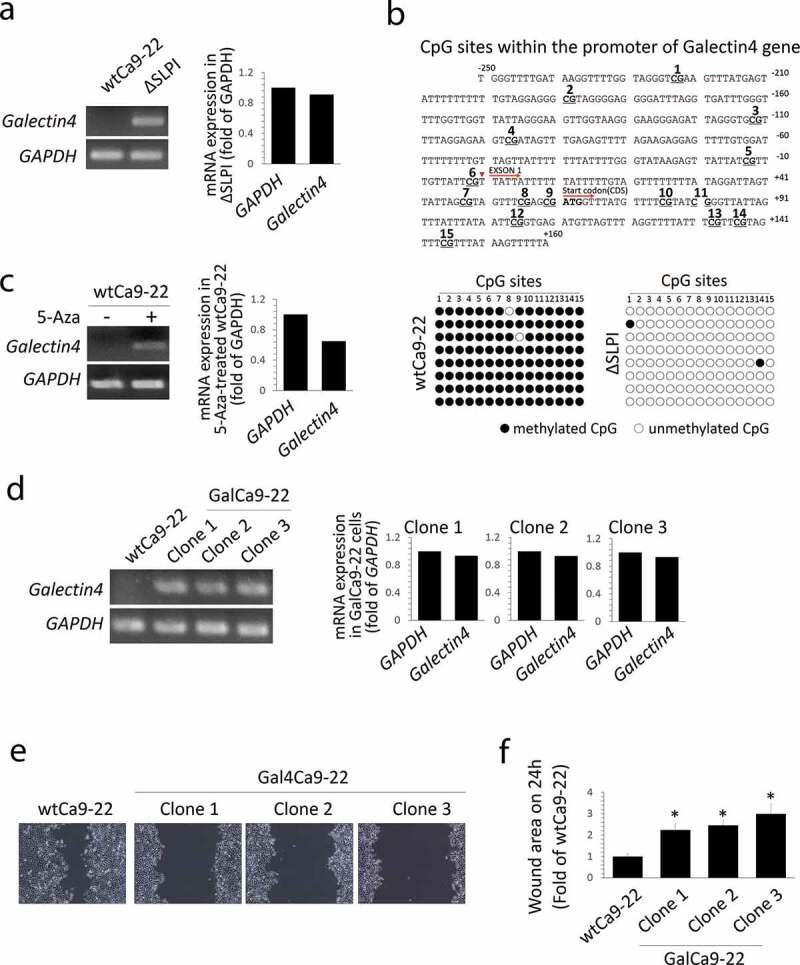
(a) mRNA expression of *Galectin4* was analyzed through RT-PCR analysis. *GAPDH* was used as an internal control. Experiments were independently repeated thrice, revealing similar results. Typical data sets are indicated in the left panel. The right graph indicates the intensity of the images of *Galectin4* and *GAPDH* in ΔSLPI cells. (b) Bisulfite sequencing analyses of the *Galectin4* gene in wtCa9-22 and ΔSLPI cells. Genomic DNA was extracted from each cell type and examined at positions −250 to +160 of the 5ʹ promoter region of *Galectin4* (relative to the transcription initiation site), containing 15 CpG sites. Methylated and unmethylated CpG sites are shown as filled and open circles, respectively. The sequences of eight bacterial clones per genomic region examined herein are shown. (c) WtCa9-22 cells were treated with 5-azacytidine (5-Aza), and *Galectin4* mRNA expression was analyzed as shown in (a). (d) *Galectin4* mRNA expression was observed in the three clones stably transfected with Galectin4 expression vectors (GalCa9-22) as shown in (a). (e) Wound healing assays were performed using the same protocol as that shown in Figure 1(a). Cells were observed using a phase-contrast microscope 24 h after the scratch ‘wound’ was inflicted. (f) Cell migration was quantified by measuring the wound areas 24 h after injury. Quantitative data are presented as mean ± standard deviation values (n = 3, *P < 0.05 vs. wtCa9-22).

In the previous microarray data [9,15], *Galectin4* mRNA was downregulated in wtCa9-22 cells; however, it was markedly upregulated in ΔSLPI cells. Therefore, we focused on this molecule. RT-PCR analysis revealed that mRNA expression of *Galectin4* was not detected in wtCa9-22 cells (Figure 4(a)). In contrast, *Galectin4* was highly expressed in ΔSLPI cells (Figure 4(a)). Bisulfite sequencing PCR assay was then performed using 8 DNA samples of each cell type to evaluate the methylation status of 15 CpG dinucleotides in the promoter region of the *Galectin4* gene (Figure 4(b) upper panel). Comparisons between wtCa9-22 and ΔSLPI cells revealed that the 15 CpG sites were highly methylated in wtCa9-22 cells compared to those in ΔSLPI cells, with a frequency of 98.3% (118/120) in the wtCa9-22 cells and 0.017% (2/120) in ΔSLPI cells (Figure 4(b) lower panel). Furthermore, treatment with demethylating agent 5-azacytidine (5-Aza) induced *Galectin4* expression in wtCa9-22 cells (Figure 4(c)). Finally, to investigate whether SLPI induces cell migration by down-regulating *Galectin4*, wtCa9-22 cells were stable-transfected with the Galectin4-expression vector. We selected three clones in the transfected cells and performed analyses for each clone. Firstly, *Galectin4* expression levels were analyzed through RT-PCR analysis. *Galectin4* mRNA was detected in the Galectin4-transfected cells (Gal4Ca9-22 cells: clone 1, 2, and 3), and their expression levels approached those of *GAPDH* and in ΔSLPI cells (Figure 4(d)). Thereafter, the migration levels of the three clones were examined through the wound healing assay. These results indicated decreased migration of the three clones from the border of the original scratch zone compared to that of wtCa9-22 cells (Figure 4(e)). These observations were supported by quantification data to determine the wound areas (figure 4(f)).

## Discussion

This study is the first to show that unlike ΔSLPI cells, wtCa9-22 cells exhibited increased migration, which was accompanied by the formation of well-developed lamellipodia and dorsal ruffles. The ability of tumor cells to migrate is an essential part of cancer metastasis and involves dynamic changes in the actin cytoskeleton [11,12]. Migrating cells rapidly assemble and turn over their actin cytoskeletons. In particular, they produce and retract protrusive actin structures, namely lamellipodia and filopodia, which lie under the plasma membrane. We previously reported [9.15] that mRNA expression levels in the Wiskott–Aldrich syndrome protein family member (*WASF)1* and *3* of the Scar/WAVE complex, a major controller of actin assembly in lamellipodia [16], in ΔSLPI cells were decreased compared to those of wtCa9-22 cells. Conceivably, SLPI seemingly accelerates the ability of cancer cells to migrate by upregulating genes associated with the organization of the actin cytoskeleton, including *WASF1* and *WASF3*.

However, cell migration is also partly regulated by cell adhesion. Intermediate filaments stabilize cell-cell adhesion complexes, which are dynamically rearranged under certain circumstances including cell migration and cancer metastasis [12]. Desmosomes are cell-cell adhesion complexes abundant in the skin and myocardium. Mutations or functional abnormalities in desmosomal structures are associated with cancer [17]. The present TEM analysis revealed that the desmosomal junctions were markedly associated with well-developed intermediate filament bundles in ΔSLPI cells compared to those in wtCa9-22 cells, suggesting the role of SLPI as a specific regulator of adhesion-related genes. Hence, we further focused on Galectin4, a molecule associated with ‘adhesion,’ which was upregulated in ΔSLPI cells compared to wtCa9-22 cells in previous microarray data [9,15]. RT-PCR data herein revealed that *Galectin4* mRNA was strongly expressed in ΔSLPI cells but not in wtCa9-22 cells. Galectin4 serves as a stabilizing component of adherens junctions [18]. Furthermore, we observed the cellular localization of Galectin4 in the cell-cell adhesion region in the ΔSLPI culture (Suppl. Fig. S1). Galectin4 downregulation was closely associated with cancer metastasis [19,20]. Wu *et al*. reported that hypermethylation of the *Galectin4* promoter is correlated with a poor prognosis among patients with urothelial carcinoma [21]. These reports support our results regarding the forced expression of Galectin4-suppressed cell migration in wtCa9-22 cells. Moreover, the *Galectin4* promoter was highly methylated in wtCa9-22 cells than in ΔSLPI cells, and 5-Aza-treatment induced *Galectin4* expression in wtCa9-22 cells. Furthermore, we confirmed that exogenous *SLPI* expression suppressed *Galectin4* expression and induced *Galectin4* promoter methylation in a human colorectal adenocarcinoma cell line HT-29, which scarcely expresses SLPI and has low migratability, as well as ΔSLPI cells (Suppl. Fig. S2). Thus, although further studies are required, our results, coupled with previous reports, suggest that SLPI suppresses the expression of certain adhesion-related genes through the induction of methylation in these gene promoters, which results in loosening of cell-cell adhesion, thus enhancing cell migration. However, our results describe how SLPI induces methylation in the promoters of these genes. Kozin et al [22]. reported that SLPI physically interacts with and phosphorylates retinoblastoma (Rb) tumor suppressor protein, this releasing Forkhead box transcriptional factor M1 (FoxM1) from the Rb-FoxM1 complex to activate transcriptional activity of FoxM1. Furthermore, activated FoxM1 induces the expression of two DNA methyltransferases *DNMT1* and *DNMT3B* genes but not *DNMT3A* gene [23]. Moreover, *DNMT1* and *DNMT3B* were upregulated in wtCa9-22 cells compared to that in ΔSLPI cells, and no significant difference was observed in *DNMT3A* mRNA between wtCa9-22 and ΔSLPI cells (Suppl. Fig. S3). Therefore, SLPI may induce DNA-methylation via FoxM1 activation in wtCa-22 cells.

In conclusion, this study reports the potential of SLPI to promote cell migration mediated through cellular microstructure regulation, including the dorsal ruffles, lamellipodia, and desmosomes. Furthermore, Galectin4 is a potential key participant in the mechanism underlying SLPI-induced cell migration.

## Materials and methods

### Cells and reagents

Ca9-22 cells were purchased from RIKEN BRC (Ibaraki, Japan). ΔSLPI cells were generated, as previously described [9]. Cells (5 × 10^4^) were seeded on 35 × 10 mm^2^ polystyrene petri dishes through physical surface treatment (Greiner Bio-One International GmbH, Kremsmünster, Austria) and were cultured in the standard medium [α-Minimal Essential Medium (α-MEM; Wako, Tokyo, Japan) containing 10% fetal bovine serum (Japan Bio Serum, Tokyo, Japan) and 1% penicillin-streptomycin (Wako)] at 37°C in a humidified atmosphere containing 5% CO_2_. For experiments using light fluorescence microscopy, cells (5 × 10^4^) were seeded on 35 × 10 mm^2^ glass bottom cell culture dishes with physical surface treatment (Greiner Bio-One International GmbH) and maintained under standard conditions. For SICM analysis, cells (2 × 10^4^ cells) were seeded on a live-cell chamber (Park Systems Corp., Suwon, Korea) and maintained under standard conditions.

### In vitro wound healing assay

Cells were cultured to confluence. At the center of the dishes, the cell monolayer was scratched with a sterile silicon tip (2 mm width) and washed with culture medium to eliminate detached cells. After the indicated periods of incubation, cells were fixed in 4% paraformaldehyde in phosphate buffer (PB; 0.1 M, pH 7.2) for 15 min, stained with 0.05% toluidine blue, and scanned using an Epson scanner GTX-890 (EPSON, Nagano, Japan). Wound areas in all dishes were measured and quantified using ImageJ software version 1.50 (National Institutes of Health, Bethesda, MD, USA).

### Scanning electron microscopy

After scratching and incubation, cells were fixed in a mixture containing 1.5% glutaraldehyde in PB for 30 min at room temperature. Fixed cells were made electron-conductive by staining with tannic acid and osmium. Cells were then dehydrated using an ascending concentration series of ethanol, transferred to isoamyl acetate (Wako), dried in a critical point dryer using CO_2_ (HCP-2, Hitachi High-technologies, Tokyo, Japan), mounted on aluminum stubs, coated with platinum–palladium in an ion sputtering device (E-1030, Hitachi Science Systems, Tokyo, Japan), and were observed using a scanning electron microscope (SEM; SU3500, Hitachi High-technologies).

### Histochemistry

After removing the culture medium, cells were fixed with 4% paraformaldehyde in PB for 30 min, rinsed thrice with PB, permeabilized with 0.01% Triton X-100 for 10 min, rinsed thrice with PBS, and stained with Alexa Fluor^TM^ 488 phalloidin (Thermo Fisher Scientific, Waltham, MA, USA) or anti-Galectin4 antibody (PROTEINTECH JAPAN, Tokyo, Japan) in PBS for 1 h at 37°C. For the phalloidin-stained cells, DAPI (4ʹ,6-diamidino-2-phenylindole, Vector Laboratories, Burlingame, CA, USA) was used to stain the nuclei. Immunofluorescence imaging of nuclei and intracellular actin molecules was performed using a confocal laser-scanning microscope (FV-300, Olympus, Tokyo, Japan). For Galectin4-stained cells, cells were subsequently incubated with a horseradish peroxidase (HRP)-conjugated secondary antibody (DAKO Japan, Tokyo, Japan) for 1 h. The cells were finally incubated with 3,3ʹ-diaminobenzidine (DAB), and nuclei were stained with hematoxylin.

### Transmission electron microscopy

Confluent cells were fixed in a mixture containing 2% paraformaldehyde and 1% glutaraldehyde in PB for 30 min. Cells were then post-fixed with 1% osmium tetroxide in PB for 30 min at 4°C. Fixed cells were dehydrated in a graded ethanol series and embedded in Epon 812 (Nisshin EM Co. Ltd., Tokyo, Japan). Ultrathin sections, approximately 70 nm thick, were cut using an ultramicrotome (Ultracut-N, Reichert-Jung, Vienna, Austria). Sections were stained with uranyl acetate and lead citrate and observed using a transmission electron microscope (TEM; H-7650, Hitachi High-technologies) at an accelerating voltage of 80 kV. The number of desmosome junctions with filaments was determined in 20 randomly selected sets of neighboring cells each from wtCa9-22 and ΔSLPI cells using TEM images.

### Gene expression analysis

A previously used microarray dataset used in this study to identify Galectin4 [9,15]. For RT-PCR analysis, total RNA was isolated from cells under standard culture conditions (un-confluent) using RNAiso Plus (Takara Bio, Shiga, Japan) in accordance with the manufacturer’s instructions. First-strand cDNA was synthesized from 1 μg of total RNA using a SuperScript III RNase H reverse transcriptase kit (Thermo Fisher Scientific). cDNA was then subjected to real-time RT-PCR using SYBR Premix Ex TaqTM II (Takara Bio) on a CFX96 Real-Time System (Bio-Rad Laboratories, Hercules, CA, USA). For RT-PCR, first-strand cDNA was synthesized as described above. PCR was performed for 40 cycles (95°C for 5 s and 68°C for 25 s). Subsequently, PCR amplicons were separated using electrophoresis on a 2% agarose gel. The primer sets used herein are described in Suppl. Table S1 online. Intensity of the exposure image was measured using Adobe Photoshop CC 2015 (Adobe, San Jose, CA, USA).

### Scanning ion conductance microscopy (SICM)

All SICM measurements were performed under ambient air conditions at 28°C. SICM images were obtained using a commercial SPM (XE-Bio system, Park Systems Corp.), wherein, the system utilizes an SPM system on the stage of an inverted optical microscope (Eclipse Ti, Nikon Corp., Tokyo, Japan). The SICM probe comprises a glass pipette filled with the electrolyte, with an Ag/AgCl electrode plugged into it. The glass pipette was fabricated from borosilicate capillaries (Narishige, Tokyo, Japan) using a CO_2_-laser-based micropipette puller (P-2000, Sutter Instruments, Novato, CA, USA); the inner and outer diameter of the pipette were approximately 100 nm and 200 nm, respectively. Images were primarily obtained in the approach-retract scanning (ARS) mode with an alternative configuration module. A resistance increase of 0.75%, with respect to basal resistance, was used to stop the approach in this experiment. To quantify the ruffle area, the SICM topographic image with cell shape and ruffles as membrane structures is constructed from 10 μm of center of the line profile on the maximum diameter in each cell image. The polynomial fitting is applied to the SICM topographic image to obtain a theoretical surface, which represents the trend of cellular morphology without ruffles. The obtained theoretical surface is then subtracted from the raw image to obtain the image of the membrane structure. The SICM images have been filtered with a mathematical procedure implemented in the free software Gwyddion. Thereafter, the cross-sectional area of each ruffle was determined from the line profile.

### Bisulfite sequencing

After seeding, cells were further cultured for 24–48 h. Thereafter, genomic DNA was purified using the PureLink^TM^ Genomic DNA Kit (Thermo Fisher Scientific) in accordance with the manufacturer’s instructions. Bisulfite conversion was performed using the EpiTect Bisulfite Kit (Qiagen, Hilden, Germany) and the converted DNA was amplified using PCR. The following primer sets were used to amplify the 437 bp promoter regions of the genes encoding *Galectin4*: forward, 5ʹ-GTT TTG ATA AGG TTT TGG T-3ʹ; reverse, 5ʹ-CCC CCA AAA TCA AAA TAA AA-3ʹ. The PCR products were cloned and expressed in *Escherichia coli* using the pGEM T-Easy vector (Promega, Madison, WI, USA), and then reverse-sequenced using the following primers: forward, 5ʹ- CCC AGT CAC GAC GTT GTA AAA CG-3ʹ; reverse, 5ʹ-CAG GAA ACA GCT ATG ACC-3ʹ.

### 5-Azacytidine treatment

We purchased 5-azacytidine (5-Aza) from Sigma-Aldrich (St. Louis, MO, USA). After cells were cultured to sub-confluence, 5-Aza was supplemented in the cultured at 10 μM. Cells were harvested after 24 h, and total RNA was extracted as described above.

### Forced expression of Galectin4 and SLPI

A PCR fragment of human *Galectin4* or *SLPI* open reading frame was cloned into pIREShyg plasmid (Takara) and the product was used as a *Galectin4*-expression or SLPI-expression vector. WtCa9-22 or HT-29 cells were seeded on 6-well plates with physical surface treatment (Greiner Bio-One International GmbH) at a density of 5 × 10^5^ cells per well and allowed to grow under standard culture conditions for 18 h. Subsequently, cells were incubated in 2 ml of α-MEM containing 6.25 μl of Lipofectamine LTX™ reagent (Thermo Fisher Scientific) and 2 μg of the expression vector. After transfection, cells with stable DNA-integration (GalCa9-22) were selected by incubation in α-MEM, containing 200 mg/ml of hygromycin (Sigma-Aldrich), 10% fetal bovine serum, and 1% penicillin-streptomycin. Twenty-four colonies were picked up and *Galectin4* or *SLPI* expression was confirmed through PCR.

### Statistical analysis

Quantitative data are presented as mean ± standard deviation. Statistical differences between two conditions were assessed using the Student’s *t*-test using the free software MEPHAS (Research Institute for Microbial Diseases, Osaka University, Osaka, Japan). All samples indicating statistical differences determined before were normally distributed. For all tests, 2-tailed tests were used, and the *P* < 0.05 was considered to indicate significant differences.

## Supplementary Material

Supplemental Material
