# Supplementary material for: SLPI facilitates cell migration by regulating lamellipodia/ruffles and desmosomes, in which Galectin4 plays an important role
Source: Cell Adh Migr. 2020 Oct 4;14(1):195–203. doi: 10.1080/19336918.2020.1829264 (PMC7553583; doi:10.1080/19336918.2020.1829264)
Supplement: Supplemental Material [file KCAM_A_1829264_SM5490.zip › Suppl_legends_rev.docx]

SLPI facilitates cell migration by regulating lamellipodia/ruffles and desmosomes, in which Galectin4 plays an important role

**Y Mizutani, D Omagari, M Hayatsu, M Nameta, K Komiyama, Y Mikami, T Ushiki**

***Supplemental Video S1****.* Wild-type (wt) Ca9-22 and secretory leukocyte protease inhibitor deleted Ca9-22 (ΔSLPI) cells were seeded on a live-cell chamber (Park Systems Corp., Suwon, Korea) and cultured in α-MEM containing 10% FBS and 1% penicillin-streptomycin at 37°C in a humidified atmosphere containing 5% CO^2^ for 24–48 h. Subsequently, SICM measurements were performed for 28.5 min. (A) wtCa9-22 cell. (B) ΔSLPI cells.

***Supplemental Video S2****.* Wild-type (wt) Ca9-22 and secretory leukocyte protease inhibitor deleted Ca9-22 (ΔSLPI) cells were seed on 35 × 10 mm^2^ polystyrene petri dishes at 2 × 10^4^ cells per dish and cultured in α-MEM containing 10% FBS and 1% penicillin-streptomycin at 37°C in a humidified atmosphere containing 5% CO^2^ for 24-48 h. Subsequently, time-lapse microscopy was performed under standard conditions using BioStudio-mini (NikonEngineering Co., LTD., Kanagawa, Japan) for the indicated culture duration. (A) wtCa9-22 cell. (B) ΔSLPI cells.

***Supplemental Figure S1****.* Secretory leukocyte protease inhibitor deleted Ca9-22 (ΔSLPI) cells were immunostained with PBS-diluted rabbit anti-human Galectin4 antibody, normal rabbit serum (control), or PBS alone (negative control), respectively. Subsequently, antibody signals were detected with 3,3’-diaminobenzidine (DAB). Nuclei were stained with haematoxylin. Arrowheads indicate Galectin4 localization on the cell-cell adhesion region.

***Supplemental Figure S2.*** (A) Human colorectal adenocarcinoma cell line HT-29 was stably transfected with SLPI-expression vectors (SLPI-HT-29). Three clones expressing *SLPI* mRNA at the same level as wtCa9-22 cells were selected, and *Galectin4* mRNA expression levels were analysed via real-time RT-PCR. Original (wild-type: wt) HT-29 cells were used as control. *Galectin4* mRNA expression levels were normalized to those of *GAPDH*. Quantitative data are presented as mean ± standard deviation values (n=3, **P*<0.05 vs. wtCa9-22). (B) Bisulphite sequencing analyses of the *Galectin4* gene in clone 1 shown in (A) and wtHT-29 cells. Genomic DNA was extracted from each cell type and examined at positions −250 to +160 of the 5’ promoter region of *Galectin4* (relative to the transcription initiation site), encompassing 15 CpG sites. Methylated and unmethylated CpG sites are indicated as filled and open circles, respectively. The sequences of eight bacterial clones per genomic region examined herein are shown.

***Supplemental Figure S3***. mRNA expression of DNA methyltransferases, *DNMT1*, *DNMT3A*, and *DNMT3B* in wild-type (wt) Ca9-22 and secretory leukocyte protease inhibitor deleted Ca9-22 (ΔSLPI) cells was analysed through real-time RT-PCR. mRNA expression of the three DNA methyltransferases were normalized to those of *GAPDH*. Quantitative data are presented as mean ± standard deviation values (n=3, **P*<0.05 vs. wtCa9-22).
