# Supplementary material for: SLPI facilitates cell migration by regulating lamellipodia/ruffles and desmosomes, in which Galectin4 plays an important role
Source: Cell Adh Migr. 2020 Oct 4;14(1):195–203. doi: 10.1080/19336918.2020.1829264 (PMC7553583; doi:10.1080/19336918.2020.1829264)
Supplement: Supplemental Material [file KCAM_A_1829264_SM5490.zip › suppl_table.docx]

SLPI facilitates cell migration by regulating lamellipodia/ruffles and desmosomes, in which Galectin4 plays an important role

**Y Mizutani, D Omagari, M Hayatsu, M Nameta, K Komiyama, Y Mikami, T Ushiki**

| ***Supplemental Table S1*.** Primers used for real-time RT-PCR and RT-PCR. | | | | |
| --- | --- | --- | --- | --- |
| gene |  | forward |  | reverse |
| *WASF1* | 5'- | GAACGTGTGGACCGTTTATCT | 5'- | GGAATAGGCAAAGTCTTGCGA |
| *WASF3* | 5'- | TGGAAGAGGTCTCACTACAGG | 5'- | TCAGAGGCGGTGGCTTATCA |
| *Galectin4* | 5'- | CGACGCTGCCTTACTACCAG | 5'- | GTAGTGCTCAGCCAGGACTATG |
| *GAPDH* | 5'- | AGTGGGGCGATGCTGGCGCTG | 5'- | TTGTCATACTTCTCATGGTTC |
