# Supplementary figures and images for: SLPI facilitates cell migration by regulating lamellipodia/ruffles and desmosomes, in which Galectin4 plays an important role
Source: Cell Adh Migr. 2020 Oct 4;14(1):195–203. doi: 10.1080/19336918.2020.1829264 (PMC7553583; doi:10.1080/19336918.2020.1829264)

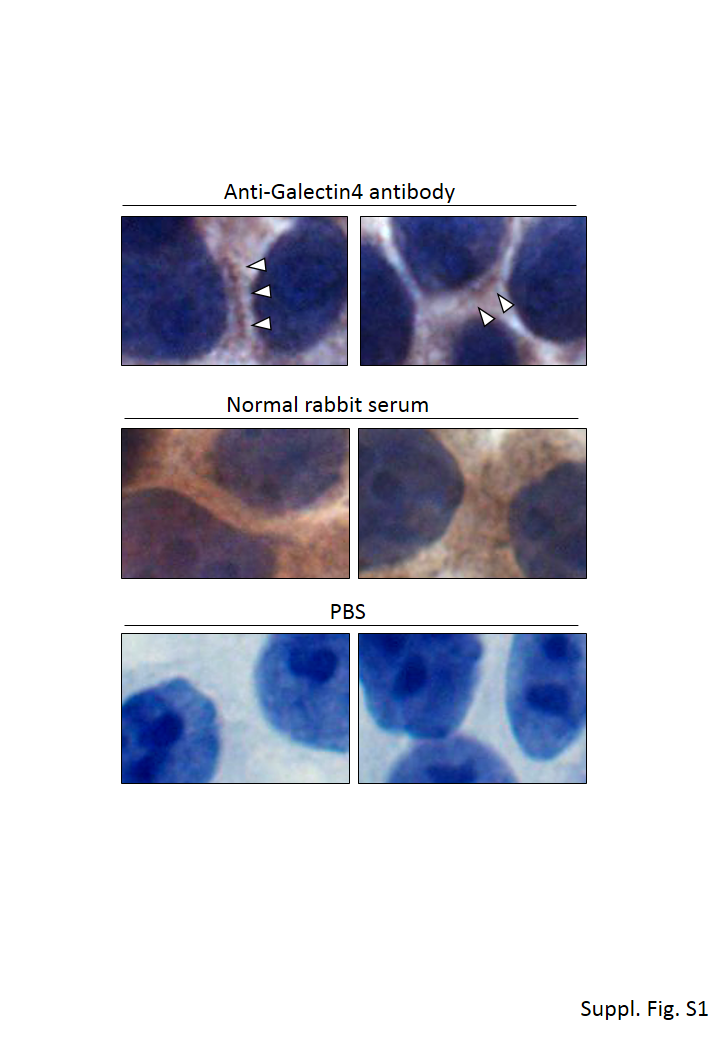

Supplement: Supplemental Material [file KCAM_A_1829264_SM5490.zip › suppl_FigS1.TIF]

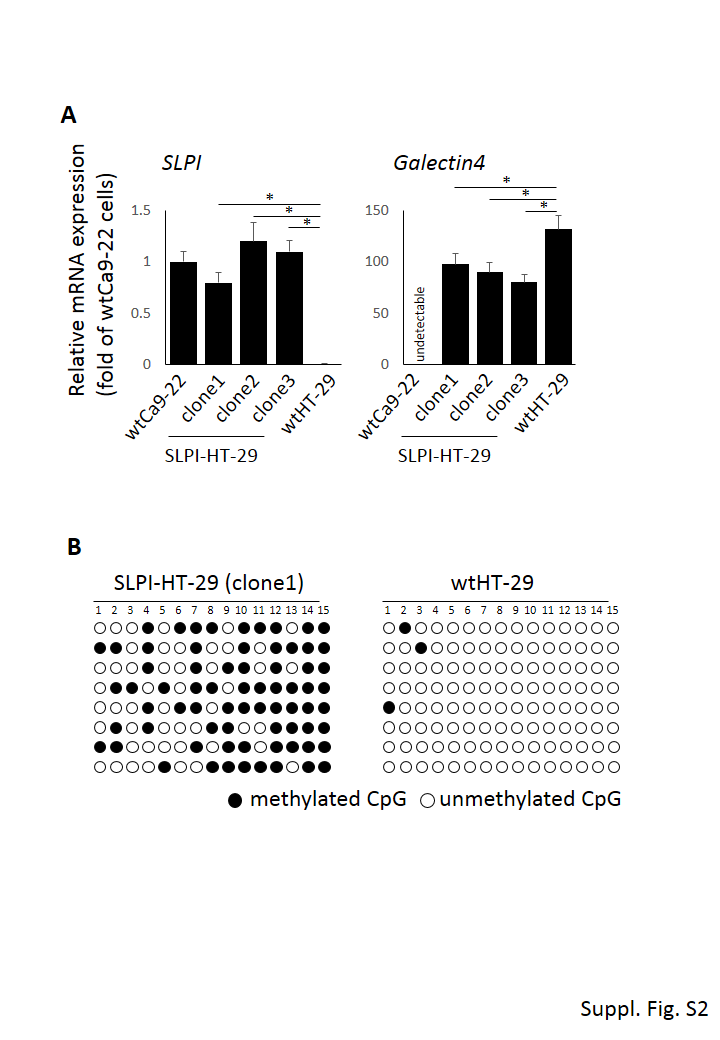

Supplement: Supplemental Material [file KCAM_A_1829264_SM5490.zip › suppl_figS2.TIF]

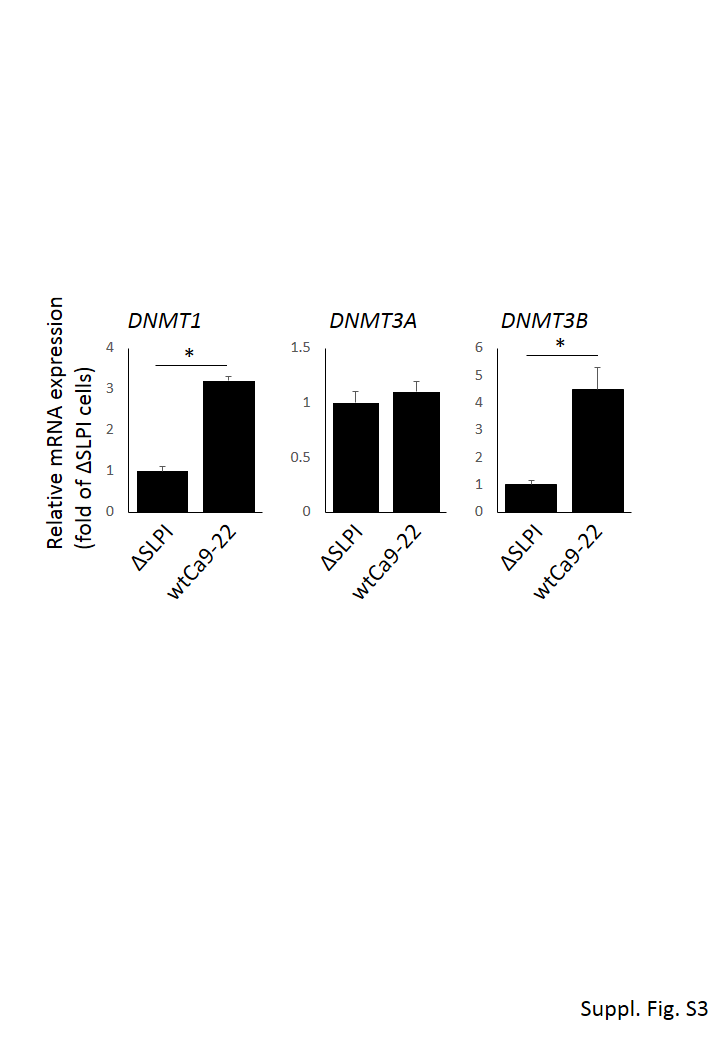

Supplement: Supplemental Material [file KCAM_A_1829264_SM5490.zip › suppl_figS3.TIF]
